# The Longitudinal Effect of *APOL1* Risk Alleles on Sickle Cell Anemia‐Associated Kidney Function

**DOI:** 10.1002/ajh.70290

**Published:** 2026-03-23

**Authors:** Sara R. Rashkin, Guolian Kang, Clifford M. Takemoto, Mitchell J. Weiss, Kenneth I. Ataga, Santosh L. Saraf, Jeffrey Lebensburger, Rima S. Zahr

**Affiliations:** ^1^ Department of Hematology St. Jude Children's Research Hospital Memphis Tennessee USA; ^2^ Department of Biostatistics St. Jude Children's Research Hospital Memphis Tennessee USA; ^3^ Center for Sickle Cell Disease University of Tennessee Health Sciences Center Memphis Tennessee USA; ^4^ Division of Hematology and Oncology University of Illinois Chicago Chicago Illinois USA; ^5^ Division of Pediatric Hematology and Oncology University of Alabama at Birmingham Birmingham Alabama USA; ^6^ Division of Pediatric Nephrology and Hypertension University of Tennessee Health Science Center Memphis Tennessee USA

**Keywords:** *APOL1*, kidney function, sickle cell anemia

## Abstract

Progressive kidney injury is a major cause of morbidity and mortality in sickle cell anemia (SCA). The high risk *APOL1* G1/G2 variants contribute to the development of kidney disease in individuals of African ancestry, including those with SCA. However, few studies have evaluated the longitudinal effect of *APOL1* variants in children and young adults. We analyzed the association of *APOL1* risk variants with kidney function in 494 individuals aged 1 to 25 in the Sickle Cell Clinical Research and Intervention Program (SCCRIP) longitudinal cohort study (clinicaltrials.gov #NCT02098863). Before age 10, *APOL1* G1/G2 alleles were not associated with time to CKD (hazard ratio [HR] = 1.87; *p* = 0.14), hyperfiltration (HR = 0.96; *p* = 0.88), or continuous eGFR (*β* = −0.0090; *p* = 0.71). However, after age 10, *APOL1* G1/G2 variants were associated with higher baseline eGFR (*β*_*APOL1*_ = 0.098; *P*_*APOL1*_ = 0.016), a steeper downward slope of eGFR over time (*β*_*APOL1*xAge_ = −0.014; *P*_*APOL1*xAge_ = 1.60 × 10^−10^), and increased odds of having an accelerated rate of eGFR decline (individual‐specific eGFR slopes in the most negative tertile; odds ratio [OR] = 2.18; *p* = 0.040). Among 111 individuals with measurements before and after 10 years of age, those with hyperfiltration before age 10 or *APOL1* risk alleles were at increased risk of having an accelerated rate of eGFR decline (OR = 2.96; *p* = 0.019). These findings support the hypothesis that genetic risk stratification and early renal surveillance can help identify patients most at risk for the progression of kidney injury.

**Trial Registration:** Clinicaltrials.gov #NCT02098863.

## 1 Introduction

Kidney damage in sickle cell anemia (SCA) results in progressive chronic kidney disease (CKD), characterized by childhood hyperfiltration, albuminuria, and subsequent decline in kidney function [1, 2]. Both the development of proteinuria/albuminuria and a rapid decline in estimated glomerular filtration rate (eGFR) have been shown to portend early CKD and mortality [3, 4]. In adults with SCA, the mean age at end‐stage kidney disease (ESKD) diagnosis (~40 years) is significantly lower than that of those without SCA, and initiation of dialysis is associated with significant increased mortality [5, 6]. Furthermore, children with SCD who develop ESKD have a longer mean time to kidney transplantation and increased overall mortality when compared to non‐SCA children with ESKD [7].

The high risk G1/G2 haplotype of *APOL1* (the gene encoding apolipoprotein L1), defined as homozygous G1 or G2 or compound heterozygous inheritance, contributes to approximately 70% of the risk of kidney disease in the general African American population [8]. Adults with high risk *APOL1* variants experience significantly faster CKD progression and a higher incidence of ESKD compared to those with wild‐type *APOL1* [8]. The incidence of new disease manifested by the development of albuminuria was reported to be significantly higher in individuals with *APOL1* G1/G2 in a non‐SCA young adult population followed over 25 years [9]. In a study of non‐SCA children with CKD and glomerular disease, while the median age of disease onset was later in children with G1/G2, the annual rate of eGFR decline was faster in children with these high risk *APOL1* variants, indicating a more aggressive form of disease [10]. Thus, *APOL1* is recognized as a “progressor” gene implicated in the advancement of CKD to ESKD in individuals without SCA [8, 11].

Our previous work and others have demonstrated that high risk *APOL1* G1/G2 variants have also been shown to contribute to kidney disease in SCA [12, 13, 14, 15, 16, 17]. In adult cohorts of patients with SCA, *APOL1* variants have been associated with elevated UACR and decreased kidney function [12, 15]. Similarly, in children with SCA, the time to development of first albuminuria is earlier in those with high risk *APOL1* variants, occurring earlier than 10 years of age [16, 17]. However, the effect of high risk *APOL1* variants on kidney function (i.e., eGFR) in SCA has not been clearly defined in children and young adults with SCA.

While it is known that *APOL1* G1/G2 variants are associated with poorer outcomes, few studies in SCA populations have examined the longitudinal effect, and none have explored the effect as children grow into adulthood, when renal manifestations progress from being subclinical to overt evidence of CKD. Understanding the effect of *APOL1* on kidney disease progression in SCA is vital for defining disease trajectory, identifying at‐risk individuals at all ages, and determining the optimal timing for prospective renoprotection [18, 19], including the use of new *APOL1* channel drug inhibitors [20, 21] under development. In this study in an updated dataset with additional patients and longer follow‐up, we assessed the effect of *APOL1* on the progression of kidney disease across the lifespan, from childhood into early adulthood.

## 2 Methods

### 2.1 Cohort Description

Individuals in our study were enrolled in the Sickle Cell Clinical Research and Intervention Program (SCCRIP), an observational cohort study with prospective follow‐up, ongoing data accrual, and retrospective collection of extensive clinical data from participants at St. Jude Children's Research Hospital (coordinating site), Methodist Comprehensive Sickle Cell Center, The Regional One Health Diggs‐Kraus Sickle Cell Clinic, and three St. Jude affiliate sites (clinicaltrials.gov #NCT02098863) [22]. For this analysis, data were collected through December 31, 2023. The study was approved by the St. Jude Children's Research Hospital Institutional Review Board, and all participants' legal guardians provided written informed consent for participation.

### 2.2 Whole Genome Sequencing

Whole genome sequencing was performed on 890 individuals, and quality control was performed to exclude (1) variants with genotype missingness > 0.01 or with Hardy–Weinberg *p* < 3 × 10^−8^; (2) individuals with genotype missingness > 0.01, excess heterozygosity (more than three standard deviations [SD] from the mean), or discordant genetic and phenotypic sex.

As our cohort was primarily comprised of African American individuals, we used the R package GENESIS [23] to perform principal component analysis (PCA) and relatedness estimation, as it is robust to known or cryptic relatedness and admixture. PCA was performed using PC‐AiR [24], which partitions the input data into related and unrelated samples, using initial relatedness estimation results from KING [25]. It then performs PCA in just the unrelated samples using only single nucleotide polymorphisms (SNPs) with minor allele frequency (MAF) > 0.01 that are in approximate linkage equilibrium (*r* ^2^ < 0.25, identified in sliding windows of 200 SNPs, shifting by 50 SNPs). Finally, it projects the principal components (PCs) into the related individuals. Kinship coefficients were then estimated using PC‐Relate [26], using ancestry representative PCs calculated from PC‐AiR to adjust for population structure.

### 2.3 Sample and Outcome Definitions

We restricted our sample to SCCRIP participants with SCA (sickle genotype HbSS or HbSβ^0^‐thalassemia) and at least one eGFR measurement between ages 1 through 25, excluding those taken during emergency and/or inpatient visits, and for whom whole genome sequencing was performed for a total sample size of 494 individuals (Figure S1).

The Chronic Kidney Disease in Children (CKiD) under 25 (U25) serum creatinine (sCr) equation [27, 28] was used to calculate eGFR. To select a corresponding height for each sCr measurement, the closest height value to the sCr measurement within 6 months, 3 years, or 6 years was chosen for ages < 18 years, 18–21 years, and 21–25 years, respectively.

As previous research has shown that early hyperfiltration prior to age 10 is associated with later changes in kidney function [29], we conducted two sets of time to event analyses (Figure S1), censored at the day before 10th and 26th birthdays, respectively. For each, individuals were classified as CKD cases if, among four consecutive measurements: (1) the first and fourth were 3–4 months apart; and (2) the first, fourth, and either second or third eGFR measurements were < 90 mL/min/1.73 m^2^. Hyperfiltration cases were defined similarly, using eGFR > 135 mL/min/1.73 m^2^ as the threshold [30]. For all outcomes, case ages were defined as the exact age at which the first instance of an event occurred, and controls were censored at the age of the last measurement. Hydroxyurea (HU) and chronic transfusion (CTXN) statuses were, separately, defined as positive if an individual was exposed prior to event occurrence or censoring.

Two sets of longitudinal analyses of continuous eGFR were also performed: before and after age 10 (Figure S1), as previous research in SCA has indicated a change in average eGFR behavior around this age [29, 31]. For these longitudinal analyses, the median of all eGFR values within each year of age was taken for each patient. Patients were included in an analysis if they had eGFR measurements taken in at least 1 year of age in the appropriate age range. To attain approximate normality within each year of age, eGFR values were log transformed. HU and CTXN statuses were defined as positive if a patient was exposed during that year of age.

*APOL1* risk status was defined as high for individuals who were homozygous for G1 (rs60910145‐G or rs73885319‐G) or G2 alleles (rs143830837‐A) or double heterozygous (heterozygous rs60910145 or rs73885319 and heterozygous rs143830837); otherwise, *APOL1* risk allele status was defined as low. Dominant allele coding was used for α‐thalassemia, where status was defined as positive if either one or two deletions were present (i.e., αα^−3.7^/αα or αα^−3.7^/αα^−3.7^).

### 2.4 Statistical Analysis

Fisher's exact test was used to compare the distribution of cases and controls for sex, α‐thalassemia, *APOL1* status, and HU and CTXN exposures. Wilcoxon rank‐sum tests were used to compare whether the median value was different between the case and control populations for age at first measurement, follow‐up time, age at end of follow‐up, number of assessments, and median interval between assessments.

Using the R package coxmeg [32], time to first event analyses evaluating the effect of *APOL1* high risk variants on CKD and hyperfiltration were conducted via Cox proportional hazards regression with a frailty model to account for relatedness among individuals and adjusting for sex, HU, CTXN, α‐thalassemia, and PC1‐5.

Longitudinal analyses were conducted using the R package GMMAT [33], using linear mixed effects models to evaluate the effect of *APOL1* high risk alleles on log(eGFR), adjusting for age, sex, HU, CTXN, α‐thalassemia, and PC1‐5, with random intercept and age terms and a kinship matrix to account for relatedness. *APOL1*‐age interaction terms were tested if both main effects were significant.

To model eGFR trajectory during adolescence and evaluate associations with differences in the rate of eGFR change over time, we estimated individual‐specific eGFR slopes after age 10 for each participant with eGFR measurements for at least 2 years of age (Figure S1). An individual's eGFR slope—the change in log(eGFR) per year after age 10—was defined as the sum of the main effect age slope term plus the person‐specific random age slope term in a multivariable linear mixed effects model for log(eGFR) using the R package lme4 [34], adjusted for the nongenetic factors of age, sex, HU, and CTXN, with random intercept and age slope terms. As we used log‐transformed eGFR in our analyses, we were unable to look at classical definitions of rapid or steeper decline of eGFR. Instead, we defined accelerated rate of eGFR decline as individuals with eGFR slopes in the bottom third across all participants, encapsulating patients with the most negative slopes. The effect of *APOL1* high risk alleles on continuous eGFR slope and accelerated rate of eGFR decline was estimated using generalized linear mixed effects models with Gaussian and binomial links, respectively, adjusting for α‐thalassemia and PC1‐5, with a random intercept and incorporating a kinship matrix. Due to the small sample size (Figure S1), Wilcoxon rank‐sum and Fisher's exact tests were used to compare the effect of either early hyperfiltration prior to age 10 or *APOL1* high risk variants on eGFR slope and accelerated rate of eGFR decline, respectively.

All analyses were conducted in R version 4.4.1. Unless otherwise specified, all tests were two‐sided, and *p* < 0.05 was considered significant.

## 3 Results

Our cohort included 494 individuals enrolled in SCCRIP with at least one eGFR measurement before age 25. Of the total 494 patients, 304 (48.03% female) had sufficient eGFR measurements to be assessed for CKD and hyperfiltration (Figure S1; Table 1). Out of 304, 46 individuals (15.13%) met criteria for CKD at a median age of 12.73 years (interquartile range [IQR] 9.42–16.13), and the remaining 258 participants were censored at a median age of 13.00 years (IQR 8.50–16.66) (Figure S1; Table 1). Similarly, 113/304 patients (37.17%) developed hyperfiltration at a median age of 4.25 years (IQR 1.24–8.77), with the remaining 191 individuals censored at a median age of 13.69 years (IQR 8.64–17.37) (Figure S1; Table 1).

**TABLE 1:** Patient characteristics table comparing those who develop CKD and hyperfiltration with controls, respectively, censored at 26th and 10th birthdays.

Censoring at 26th birthday								
	CKD				Hyperfiltration			
	Total (*N* = 304)	Cases (*N* = 46)	Controls (*N* = 258)	*p* ^a^	Total (*N* = 304)	Cases (*N* = 113)	Controls (*N* = 191)	*p* ^a^
Female, *N* (%)	146 (48.03)	21 (45.65)	125 (48.45)	0.75	146 (48.03)	48 (42.48)	98 (51.31)	0.15
Age at first eGFR, median (IQR)	1.69 (1.17–4.20)	3.56 (2.02–6.39)	1.51 (1.14–3.55)	8.92 × 10^−6^	1.69 (1.17–4.20)	1.66 (1.17–4.25)	1.84 (1.17–4.13)	0.87
Age at end of follow‐up, median (IQR)	16.50 (11.41–20.74)	18.05 (15.04–20.01)	16.17 (11.03–20.83)	0.27	12.98 (8.11–19.15)	8.50 (5.39–12.55)	16.73 (11.03–22.44)	3.31 × 10^−18^
Follow‐up time (years)^b^, median (IQR)	13.00 (8.71–16.53)	12.73 (9.42–16.13)	13.00 (8.50–16.66)	0.89	9.82 (4.61–15.12)	4.25 (1.24–8.77)	13.69 (8.64–17.37)	1.96 × 10^−24^
Number of eGFR assessments, median (IQR)	57.00 (35.75–90.00)	99.00 (74.50–121.00)	52.00 (32.00–82.00)	3.98 × 10^−9^	57.00 (35.75–90.00)	70.00 (44.00–129.00)	52.00 (32.50–79.00)	0.00025
Median interval between eGFR assessments (years), median (IQR)	0.15 (0.082–0.18)	0.15 (0.077–0.17)	0.15 (0.092–0.19)	0.34	0.15 (0.082–0.18)	0.13 (0.077–0.16)	0.16 (0.12–0.19)	3.63 × 10^−5^
HU exposure, *N* (%)	278 (91.45)	40 (86.96)	238 (92.25)	0.25	252 (82.89)	69 (61.06)	183 (95.81)	1.28 × 10^−14^
CTXN exposure, *N* (%)	124 (40.79)	26 (56.52)	98 (37.98)	0.023	113 (37.17)	45 (39.82)	68 (35.60)	0.46
*α*‐thalassemia *α* ^−3.7^ deletion^c^, *N* (%)	90 (29.61)	15 (32.61)	75 (29.07)	0.60	90 (29.61)	27 (23.89)	63 (32.98)	0.12
*APOL1* high risk positive^d^, *N* (%)	38 (12.50)	7 (15.22)	31 (12.02)	0.63	38 (12.50)	15 (13.27)	23 (12.04)	0.86

Censoring at 10th birthday								
	CKD				Hyperfiltration			
	Total (*N* = 194)	Cases (*N* = 8)	Controls (*N* = 186)	*p* ^a^	Total (*N* = 194)	Cases (*N* = 81)	Controls (*N* = 113)	*p* ^a^
Female, *N* (%)	98 (50.52)	5 (62.50)	93 (50.00)	0.72	98 (50.52)	36 (44.44)	62 (54.87)	0.19
Age at first eGFR, median (IQR)	1.44 (1.11–2.34)	1.37 (1.14–2.03)	1.44 (1.11–2.36)	0.88	1.44 (1.11–2.34)	1.62 (1.17–2.83)	1.30 (1.10–2.03)	0.039
Age at end of follow‐up, median (IQR)	9.76 (8.38–9.93)	7.13 (5.59–8.11)	9.78 (8.70–9.94)	0.00083	8.55 (5.31–9.81)	5.63 (3.21–7.95)	9.76 (8.57–9.93)	5.83 × 10^−19^
Follow‐up time (years)^b^, median (IQR)	7.47 (5.56–8.42)	4.92 (2.57–6.53)	7.57 (5.79–8.45)	0.0062	5.99 (2.64–7.94)	2.85 (1.07–4.82)	7.73 (5.87–8.47)	5.70 × 10^−19^
Number of eGFR assessments, median (IQR)	35.00 (24.00–46.00)	50.50 (25.50–85.50)	35.00 (24.00–44.00)	0.11	35.00 (24.00–46.00)	39.00 (28.00–49.00)	29.00 (23.00–41.00)	0.00078
Median interval between eGFR assessments (years), median (IQR)	0.15 (0.081–0.17)	0.077 (0.077–0.16)	0.15 (0.083–0.17)	0.15	0.15 (0.081–0.17)	0.11 (0.077–0.15)	0.15 (0.096–0.19)	0.0022
HU exposure, *N* (%)	166 (85.57)	5 (62.50)	161 (86.56)	0.092	153 (78.87)	52 (64.20)	101 (89.38)	3.14 × 10^−5^
CTXN exposure, *N* (%)	67 (34.54)	5 (62.50)	62 (33.33)	0.13	61 (31.44)	27 (33.33)	34 (30.09)	0.64
*α*‐thalassemia *α* ^−3.7^ deletion^c^, *N* (%)	57 (29.38)	0 (0)	57 (30.65)	0.11	57 (29.38)	22 (27.16)	35 (30.97)	0.63
*APOL1* high risk positive^d^, *N* (%)	27 (13.92)	3 (37.50)	24 (12.90)	0.084	27 (13.92)	13 (16.05)	14 (12.39)	0.53

Abbreviations: CKD = chronic kidney disease; CTXN = chronic transfusion; eGFR = estimated glomerular filtration rate; HU = hydroxyurea; IQR = interquartile range.

^a^ Two‐sided Fisher's exact test was used for sex, HU exposure, CTXN exposure, and genetic variants. A two‐sided Wilcoxon rank sum test was used for all other variables.

^b^ Follow‐up time defined as years between first eGFR measurement and event or censoring.

^c^ *α*‐thalassemia *α* ^−3.7^ deletion = either *α*/*α* ^−3.7^ or *α* ^−3.7^/*α* ^−3.7^.

^d^ *APOL1* high risk positive = homozygous G1 or G2 or double heterozygous.

Among all 304 individuals, 38 (12.50%) had high risk *APOL1* (CKD: 7 cases [15.22%] and 31 controls [12.02%]; hyperfiltration: 15 cases [13.27%] and 23 controls [12.04%]), and *APOL1* status was not associated with either CKD (*p* = 0.63) or hyperfiltration (*p* = 0.86) (Table 1). Similarly, in multivariable Cox proportional hazards models adjusting for *APOL1* high risk status, sex, α‐thalassemia, HU, CTXN, and PC1‐5, the risk for neither CKD (HR = 1.87; *p* = 0.14; Figure S2A) nor hyperfiltration (HR = 0.96; *p* = 0.88; Figure S2B) was associated with *APOL1* variants.

### 3.1 Before Age 10

Prior to 10 years of age, 194 individuals (50.52% female) had sufficient eGFR measurements to assess CKD and hyperfiltration (Figure S1; Table 1). Only 8/194 patients (4.12%) met criteria for CKD at a median age of 4.92 years (IQR 2.57–6.53), and the remaining 186 individuals were censored at a median age of 7.57 years (IQR 5.79–8.45) (Figure S1; Table 1). In contrast, out of 194 participants, hyperfiltration occurred in 81 patients (41.75%) at a median age of 2.85 years (IQR 1.07–4.82), and the remaining 109 individuals were censored at a median age of 7.73 years (IQR 5.87–8.47) (Figure S1; Table 1).

Among all 194 individuals, 27 (13.92%) had high risk *APOL1* (CKD: Three cases [37.50%] and 24 controls [12.90%]; hyperfiltration: 13 cases [16.05%] and 14 controls [12.39%]), and *APOL1* status was associated with neither CKD (*p* = 0.084) nor hyperfiltration (*p* = 0.53) (Table 1). Due to the small number of CKD cases, a multivariable analysis was not conducted (Figure S2C). However, in multivariable models adjusted for sex, α‐thalassemia, HU, CTXN, and PC1‐5, *APOL1* high risk variants were not associated with time to development of hyperfiltration (HR = 1.27; *p* = 0.47; Figure S2D).

**FIGURE 1:**
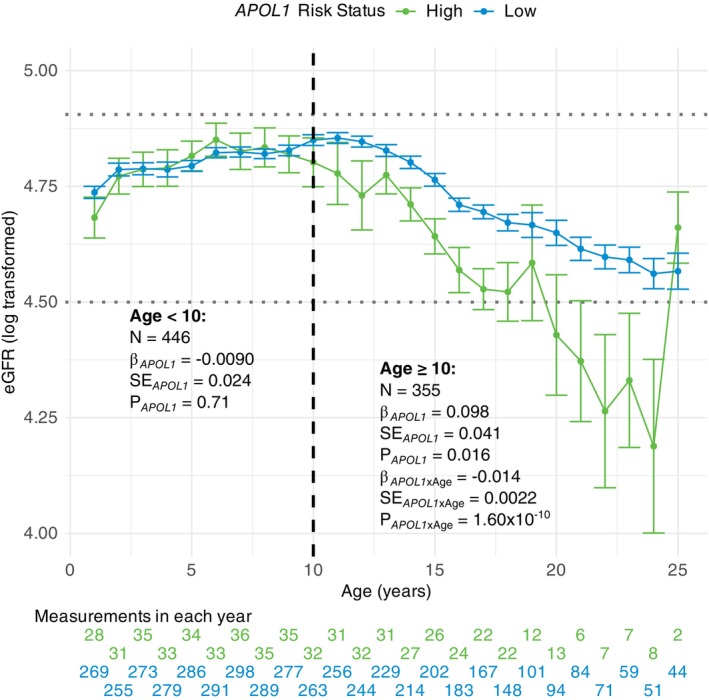
Estimated glomerular filtration rate (eGFR) by age and *APOL1* risk status. For each year of age, the average log‐transformed eGFR across all measurements at that age is plotted with bars indicating the standard error for individuals with (green) and without (blue) high risk *APOL1* variants. The number of measurements included at each age for both *APOL1* risk statuses are listed below the plot. A black dashed line indicates age 10, which stratifies our analysis. Gray dotted lines indicate the thresholds for hyperfiltration (eGFR > 135 mL/min/1.73 m^2^) and chronic kidney disease (eGFR < 90 mL/min/1.73 m^2^) after the same log transformation (4.91 and 4.50, respectively). Regression coefficients (*β*), standard errors (SE), and *P* were estimated using linear mixed regression models adjusted for age, sex, hydroxyurea exposure, chronic transfusion exposure, *α*‐thalassemia, and the first five principal components, with random intercept and slope terms and a kinship matrix to account for relatedness between individuals. For age ≥ 10, the *APOL1*‐age interaction was also included. [Color figure can be viewed at wileyonlinelibrary.com]

For longitudinal models, there were 2817 eGFR measurements for 446 individuals (median 7 measurements per patient) (Figure S1; Figure 1). Multivariable linear mixed regression for eGFR (log‐transformed) was conducted, incorporating *APOL1* high risk status, age, sex, α‐thalassemia, HU, CTXN, and PC1‐5. High risk *APOL1* variants were not associated with eGFR (𝛽 = −0.0090; *p* = 0.71) (Figure 1).

### 3.2 After Age 10

Considering only data in ages ≥ 10, there were 2712 eGFR measurements for 355 individuals (median 8 measurements per patient) (Figure S1; Figure 1). Multivariable models were constructed incorporating *APOL1* high risk status, age, sex, α‐thalassemia, HU, CTXN, and PC1‐5 as well as the *APOL1‐age* interaction. *APOL1* variants were associated with an increase in baseline eGFR (𝛽_*APOL1*_ = 0.098; *P*_*APOL1*_ = 0.016) and a faster rate of eGFR decline with age (𝛽_*APOL1* × Age_ = −0.014; *P*_*APOL1* × Age_ = 1.60 × 10^−10^) (Figure 1).

**FIGURE 2:**
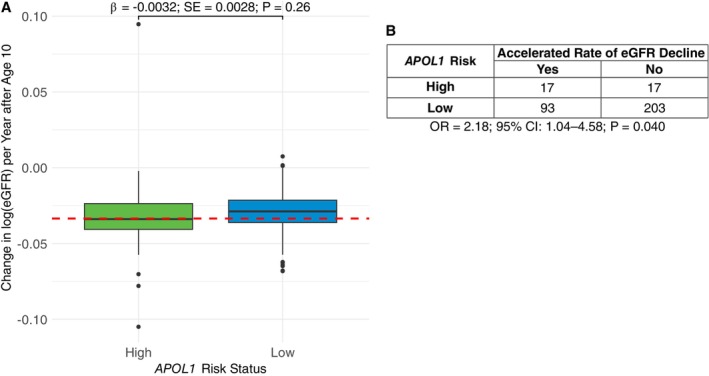
Kidney function after age 10 by the *APOL1* high risk status. (A) Comparison of estimated glomerular filtration rate (eGFR) slope, measured as the change in log(eGFR) per year after age 10 by the presence of *APOL1* high risk (green) compared to low risk (blue). The effect (*β*), standard error (SE), and *P* for the association of *APOL1* high risk alleles on continuous eGFR slope was estimated using linear mixed effects regression, adjusting for α‐thalassemia, PC1‐5, a random intercept, and kinship matrix. Accelerated rate of eGFR decline, defined as an eGFR slope in the lower tertile, capturing individuals with the most negative slope, is indicated by the dashed red line. (B) Accelerated rate of eGFR decline by presence of either *APOL1* risk variants. Odds ratio (OR), 95% confidence interval (CI), and *P* were computed using generalized linear mixed effects regression with binomial link, adjusting for the same covariates as in (A). [Color figure can be viewed at wileyonlinelibrary.com]

Among the 330 individuals with at least two eGFR measurements at age ≥ 10, 110 (33.33%) were classified as having an accelerated rate of eGFR decline (Figure S1; Figure 2). While *APOL1* G1/G2 was not associated with continuous eGFR slope (𝛽 = −0.0032; *p* = 0.26; Figure 2A), high risk *APOL1* was associated with an increased odds of an accelerated rate of eGFR decline (OR = 2.18; *p* = 0.040; Figure 2B).

**FIGURE 3:**
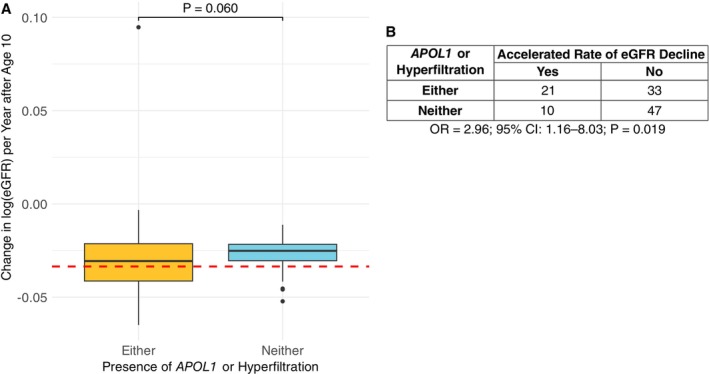
Kidney function after age 10 by the presence of either *APOL1* risk or early hyperfiltration. (A) Comparison of estimated glomerular filtration rate (eGFR) slope, measured as the change in log(eGFR) per year after age 10 by the presence of either *APOL1* high risk variants or early hyperfiltration before age 10 (yellow) compared to neither (light blue). Wilcoxon rank‐sum test was used to evaluate if the medians of the two groups was the same. Accelerated rate of eGFR decline, defined as an eGFR slope in the lower tertile of all patients, capturing individuals with the most negative slopes, is indicated by the dashed red line. (B) Accelerated rate of eGFR decline by presence of either *APOL1* risk variants or early hyperfiltration. Odds ratio (OR), 95% confidence interval (CI), and *P* were computed using Fisher's exact test. [Color figure can be viewed at wileyonlinelibrary.com]

As previous research has shown that early hyperfiltration prior to age 10 is associated with later changes in kidney function [29], we also considered early hyperfiltration (prior to age 10) as well as *APOL1* risk allele status. Among the 111 individuals with sufficient data before and after age 10 to include in the analysis (Figure S1; Figure 3), 54 (48.65%) had either early hyperfiltration or high risk *APOL1*, and 31/111 individuals (27.93%) were classified as having an accelerated rate of eGFR decline (Figure 3). Although the difference was not significant, eGFR slopes trended more steeply negative among patients with either early hyperfiltration or high risk *APOL1* (Figure 3A). However, individuals with either hyperfiltration or *APOL1* G1/G2 variants had increased odds for an accelerated rate of eGFR decline (OR = 2.96; *p* = 0.019; Figure 3B).

## 4 Discussion

Kidney disease in individuals with SCA is characterized by increased morbidity and early mortality. Therefore, understanding the risk factors for kidney disease, particularly genomic risk factors, is crucial for identifying individuals at higher risk. At present, no therapies have been proven to prevent the progression from chronic kidney disease to end‐stage kidney disease. Therefore, understanding which risk factors, including genomic risk factors, contribute to the early progression to CKD and ESKD is critical to developing intervention strategies. To our knowledge this is the first study to evaluate the longitudinal effect of *APOL1* high risk G1/G1 alleles on eGFR and eGFR slope over time in kidney function in children and young adults with SCA. Our study highlights the importance of high risk *APOL1* on kidney disease progression as we demonstrate that, in individuals with G1/G2 variants, decreased kidney function (measured by continuous eGFR) is seen after age 10 years.

High risk *APOL1* alleles have been previously associated with kidney function in both SCA cohorts and the general population. In an adult SCA cohort, high risk *APOL1* G1/G2 has been linked to the progression of CKD [15]. Another study in adults with SCA in France found that high risk *APOL1* was associated with worsening albuminuria and decreased kidney function in an age‐dependent manner [35]. In a pediatric cohort assessing the impact of *APOL1* on non‐SCA‐associated kidney disease, the median age of disease onset was older in children with *APOL1* G1/G2 variants but was a more aggressive form of disease, characterized by a steeper decline in eGFR [10]. While *APOL1* status was not associated with time to hyperfiltration or CKD in our analysis, *APOL1* variants were associated with higher baseline eGFR and a significantly steeper rate of decline after age 10. This pattern may reflect an initial early and severe hyperfiltration phase followed by accelerated glomerular injury consistent with *APOL1*‐associated diseases described in other populations [36, 37, 38]. Further, as novel therapeutics are being developed to target *APOL1*‐associated kidney injury, this study identifies an age after which intervention trials could begin in patients with sickle cell disease.

In addition to identifying an association between *APOL1* high risk variants and decline in kidney function after age 10, we also identified an association between hyperfiltration before age 10 and an accelerated decline in kidney function after age 10. It is well‐established that hyperfiltration significantly contributes to glomerular basement membrane injury, albuminuria, and the progression of kidney disease in non‐SCA diseases including diabetes, obesity, and hypertension [39]. Hyperfiltration is a common finding in SCA, with prevalence rates ranging between 16% and 80% in children and young adults [29, 40, 41, 42, 43]. In our study, the hyperfiltration prevalence supported these reports, occurring in 41.75% of patients before age 10, while CKD was relatively rare in this age group (4.12%). Early hyperfiltration between the ages of 4–10 years has been shown to be associated with an increased probability to develop persistent albuminuria in children [29]. Though our data did not explore the development of albuminuria, we have similarly demonstrated a peak in eGFR around age 10 followed by a subsequent decline in kidney function. Combined, these data suggest that hyperfiltration may precede measurable decline in kidney function and could serve as an early biomarker of renal stress, underscoring the importance of hyperfiltration monitoring in children with and without high risk *APOL1*. Future studies of hyperfiltration will need to evaluate the timing of onset, the magnitude, and duration of hyperfiltration on the progression of CKD and ESKD. This information can inform the monitoring and endpoints of future therapeutic studies of GFR changes in early childhood.

Rapid decline in kidney function is associated with increased morbidity and mortality in adults with CKD [44, 45]. Given the risk for early ESKD in SCA, identifying individuals at increased risk for kidney function decline is important [6, 7]. In our study, individuals with *APOL1* high risk alleles had increased odds of an accelerated rate of eGFR decline after age 10, reinforcing the potential importance of utilizing *APOL1* alleles to risk stratify patients. Individuals possessing these variants would likely benefit from early screening and potential intervention, though further work, including prospective intervention studies, is required to fully assess the benefit of such risk stratification in the context of SCA‐related kidney disease.

Our study had several limitations, primarily due to data sparsity. In particular, while we were able to identify that individuals with either hyperfiltration prior to age 10 or *APOL1* risk variants were more likely to have a decline in kidney function after age 10, due to sample size limitations, we were unable to adjust for other covariates of interest or fully evaluate the interplay and potential interaction between early hyperfiltration and *APOL1* risk status on later renal function. Additionally, we limited our cohort analysis to up to 26 years of age due to data sparsity and limited agreement between pediatric and adult eGFR formulas at age thresholds. To fully elucidate the effect of *APOL1* throughout the lifespan, we need both more robust adult data as well as earlier screening to highlight genetic and biomarker risk factors indicative of poor outcomes. As our cohort ages and more clinical data are collected at all ages, we will be able to further assess changes in kidney function and the effect of *APOL1* with corresponding kidney function decline with progression of CKD. We also lacked a formal replication cohort, so further validation is needed to confirm our results. Finally, we were unable to adjust our models to incorporate the use of medication including angiotensin‐converting enzyme inhibitors and angiotensin receptor blockers due to limited use within our cohort.

Due to the progressive nature of rapid decline to ESKD and its associated high mortality, it is critical that clinicians can identify at‐risk patients and ensure appropriate intensive screening approaches are implemented. The long‐term goal remains to develop appropriate risk‐targeted renoprotective intervention studies that can prevent the development and progression to ESKD. As no current therapies have been proven to prevent progression to ESKD in patients living with SCA, identifying a very high‐risk ESKD population may allow patients to consider curative and transformative intervention approaches earlier in life.

This study highlights that patients with SCA who are older than age 10 and have high risk *APOL1* variants experience a more rapid decline in eGFR. Our prior work identified that patients with high risk *APOL1* experience earlier development of albuminuria [16, 17]. Incorporating genetic screening in addition to traditional kidney screening with eGFR represents a novel approach to identifying higher risk patients. With the important development of *APOL1* mediated interventions for other diseases, it is plausible to develop intervention studies for patients living with SCA who have early CKD and high risk alleles. Finally, the SCCRIP cohort will continue to evaluate the utility of incorporating a polygenic risk stratification approach to screening and monitoring the lifetime progression from hyperfiltration to CKD and ESKD.

## Funding

The project described was supported by the National Institutes of Health through K23HL157554 (R.S.Z., G.K.). The content is solely the responsibility of the authors and does not necessarily represent the official views of the National Institutes of Health.

## Ethics Statement

The study was approved by the St. Jude Children's Research Hospital Institutional Review Board.

## Consent

All participants' legal guardians provided written, informed consent for participation.

## Conflicts of Interest

S.R.R. and G.K. have nothing to disclose. C.M.T. is a site‐PI for SCD trials sponsored by Novonordisk and Pfizer. M.J.W. is a consultant for Sanofi. K.I.A. has served on advisory boards or as a consultant for Octapharma, Novo Nordisk, Agios Pharmaceuticals, Pfizer, Sanofi, Fulcrum Therapeutics, GSK, Novartis, and Protagonist Therapeutics; serves on the Data Monitoring Committee for Vertex; and is a member of the Board of Trustees for Vitalant. S.L.S. has served on advisory boards or as a consultant for Agios, BEAM therapeutics, Forma/Novo Nordisk, Novartis, Global Blood Therapeutics/Pfizer, Chiesi, and Fulcrum. J.L. is a consultant for Novartis, Agios, and Pfizer related to sickle cell kidney studies. R.S.Z. serves as a consultant for Pfizer Inc.

## Supporting information


**Figure S1:** Flowchart of analysis.
**Figure S2:** Kaplan–Meier plots for time to CKD and hyperfiltration by *APOL1* risk status.

## Data Availability

Whole‐genome sequencing data are available via dbGaP (phs002470.v1.p1) and the St. Jude Cloud (https://platform.stjude.cloud/data/cohorts; accession no.: SJC‐DS‐1006) upon request and subsequent approval by the SGP steering committee.
