# Supplementary material for: The Longitudinal Effect of APOL1 Risk Alleles on Sickle Cell Anemia‐Associated Kidney Function
Source: Am J Hematol. 2026 Mar 23;101(6):1341–50. doi: 10.1002/ajh.70290 (PMC13139885; doi:10.1002/ajh.70290)

The longitudinal effect of APOL1 risk alleles on sickle cell anemia-associated kidney function
Rashkin SR, et al.

Supplementary Figure 1. Flowchart of analysis.

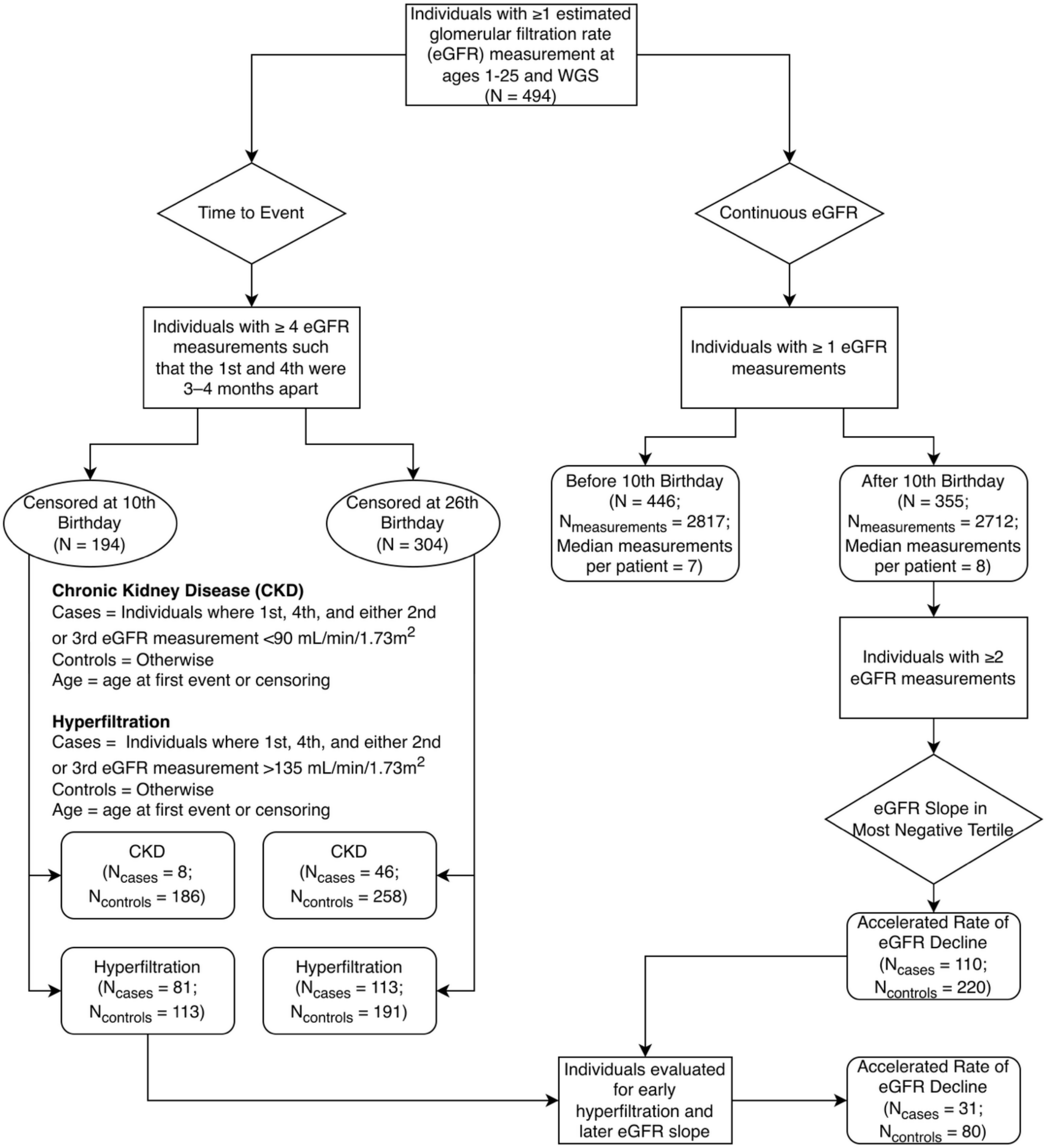

Supplementary Figure 2. Kaplan-Meier plots for time to CKD and hyperfiltration by *APOL1* risk status. Cumulative incidence of CKD (A and C) and hyperfiltration (B and D) censored at 26th (A and B) and 10th (C and D) birthdays by *APOL1* risk status. Hazard ratios (HR), 95% confidence intervals (CI), and P-values were estimated using a Cox frailty model to account for relatedness structure and adjusted for sex, hydroxyurea exposure, chronic transfusion exposure, α-thalassemia, and the first five principal components. Due to infrequent incidence of CKD when censored at the 10th birthday (N_{cases} = 8), multivariable regression was not determined (ND) for this outcome.

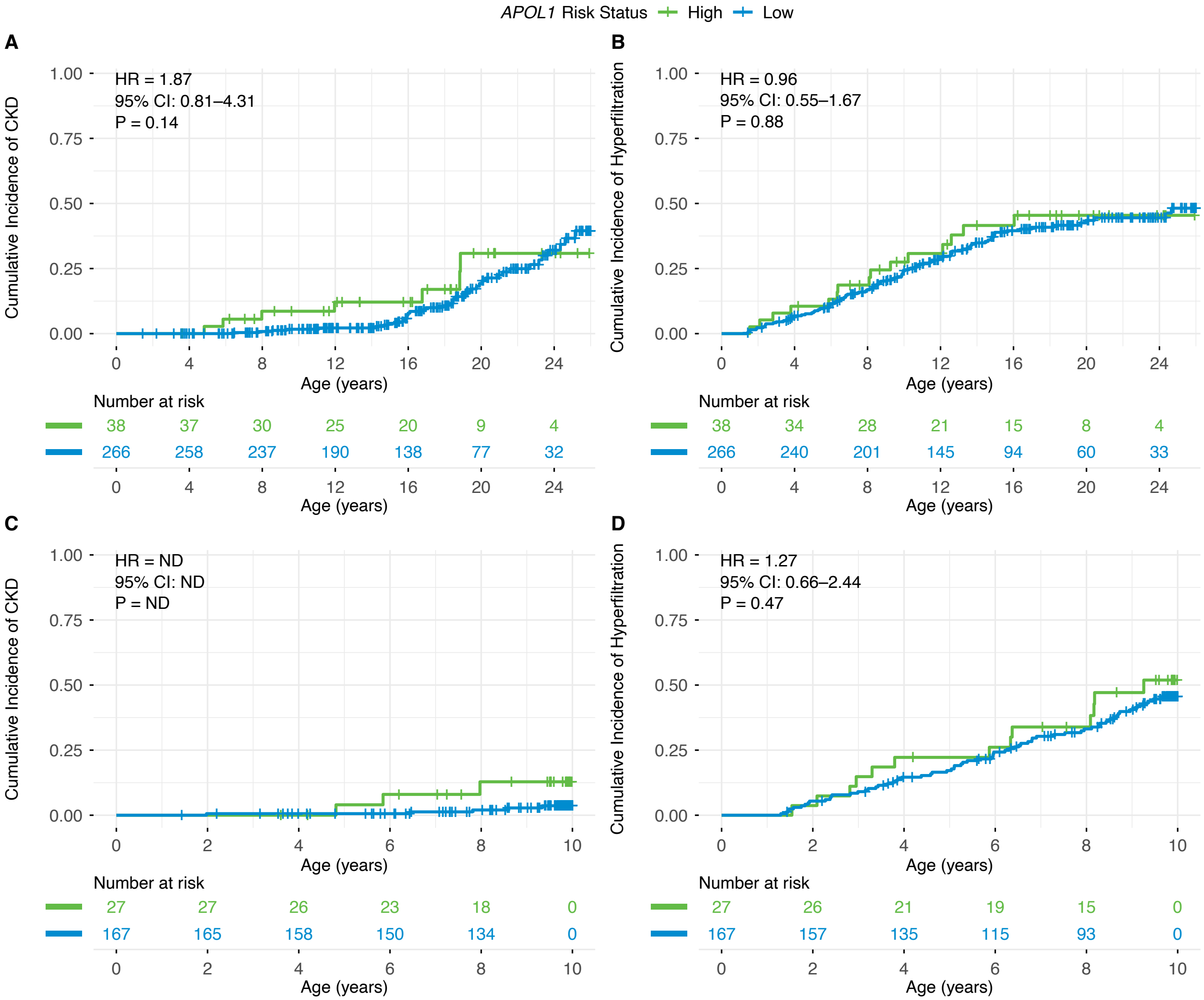

Supplement: Supplementary file 1 — Figure S1: Flowchart of analysis. Figure S2: Kaplan–Meier plots for time to CKD and hyperfiltration by APOL1 risk status. [file AJH-101-1341-s001.pdf]
